# Utilising fax to PDF technology to increase security, confidentiality, protocol adherence, follow-up and ease of monitoring in multicentre randomised trials

**DOI:** 10.1186/1745-6215-14-S1-P56

**Published:** 2013-11-29

**Authors:** Martin Dennis, Rustam Al-Shahi Salman, Gillian Mead, Christopher Matthews, Jonathan Drever, David Perry

**Affiliations:** 1 University of Edinburgh, Edinburgh, UK

## 

In the ongoing multicentre FOCUS (http://www.focustrial.org.uk) and RESTART (http://www.RESTARTtrial.org) trials we have provided our centres with a dedicated trial specific Fax number. However, instead of printing out the document, a fax modem converts it to a pdf which is automatically emailed into our trial inbox. This attachment is automatically stripped off the email and inserted into the trial IT system inbox. Within the trial IT system the pdfs can be viewed, categorised, e.g. consent form, confirmation of IMP dispensing, delegation log; attributed to a centre or participant and then quality assured by our staff. If a document is incomplete, unsigned or the wrong version, immediate action can be taken to rectify the situation. Each type of document has a specific set up quality assurance questions associated with it.

The reception of pdf's is more easily secured and made confidential than documents being printed off by a fax machine. The pdf of documents are stored in a "virtual site file" which can be accessed centrally by our monitor, as well as by the centre. Our monitor can check on the most important trial procedures, e.g. cross referencing signatures on consent forms with delegation logs and training certificates, without travelling to a centre. Also, it facilitates our central follow-up via GPs. We send a signed copy of the consent form to the GPs, along with our questionnaires, as proof of the patients' agreement to them giving us their information. This has greatly reduced the work involved in follow-up.

